# Validation of Reference Genes for the Determination of Platelet Transcript Level in Healthy Individuals and in Patients with the History of Myocardial Infarction

**DOI:** 10.3390/ijms14023456

**Published:** 2013-02-06

**Authors:** Katalin S. Zsóri, László Muszbek, Zoltán Csiki, Amir H. Shemirani

**Affiliations:** 1 Clinical Research Center, University of Debrecen, Medical and Health Science Center, Debrecen 4032, Hungary; E-Mails: zskatika@gmail.com (K.S.Z.); muszbek@med.unideb.hu (L.M.); 2 Thrombosis and Haemostasis Research Group of the Hungarian Academy of Sciences, University of Debrecen, Medical and Health Science Center, Debrecen 4032, Hungary; 3 Department of Internal Medicine, University of Debrecen, Medical and Health Science Center, Debrecen 4032, Hungary; E-Mail: csikiz@gmail.com

**Keywords:** transcript level, normalization, platelet, reference gene, RT-qPCR

## Abstract

RT-qPCR is the standard method for studying changes in relative transcript level in different experimental and clinical conditions and in different tissues. No validated reference genes have been reported for the normalization of transcript level in platelets. The very low level of platelet RNA and the elimination of leukocyte contamination represented special methodological difficulties. Our aims were to apply a simple technique to separate platelets for transcript level studies, and select the most stable reference genes for platelets from healthy individuals and from patients with the history of myocardial infarction. We developed a simple, straightforward method of platelet separation for RNA isolation. Platelet activation was inhibited by using acid-citrate-dextrose for anticoagulation and by prostaglandin E1. Leukocyte contamination was eliminated by three consecutive centrifugations. Samples prepared by this method were free of leukocytes, showed no inhibition in PCR reaction and no RNA degradation. The assay demands low blood volume, which complies with the requirements of everyday laboratory routine. Seventeen potential reference genes were investigated, but eight of them were excluded during optimization. The stability of the remaining genes, *EEF2*, *EAR*, *ACTB*, *GAPDH*, *ANAPC5*, *OAZ1*, *HDGF*, *GNAS*, and *CFL1*, were determined by four different descriptive statistics. *GAPDH*, *GNAS*, and *ACTB* were shown to be the most stable genes in platelets of healthy individuals, while *HDGF*, *GNAS*, and *ACTB* were the most stable in platelets of patients with the history of myocardial infarction. The results confirm that data normalization needs assessment of appropriate reference genes for a particular sample set.

## 1. Introduction

Reverse transcription quantitative real-time PCR (RT-qPCR) is, at present, the most sensitive method for the detection and quantitation of low abundance mRNAs [1]. It is crucial to control for variation between samples when measuring mRNA expression. One approach is to normalize to total RNA. This approach needs a reliable RNA quantification method and fails to take into account the variability in reverse transcription and other steps of the measurement [2]. The use of reference genes (RGs) as an internal control is the most common approach for data normalization [3]. On the other hand, the use of a single internal RG for normalization could lead to relatively large errors [4]. RGs used for the quantification of mRNA expression could vary with tissue type as well as with physiological, pathological and experimental conditions, and their validation and optimization for accurate and reproducible quantitation is essential [5]. Previous reports on platelet mRNA expression used conventional RGs [6,7], but details concerning their selection, validation, and investigation of stable expression were not provided.

Circulating platelets contain an exceptionally small amount of megakaryocyte derived mRNA. Low mRNA concentration and contamination with leukocytes are two main hindrances of platelet transcript level studies. To decrease the volume of whole blood required for single individual transcript level studies and to amplify the starting platelet mRNA, different amplification strategies have been used [8]. Leukocyte contamination was decreased by filtration and magnetic leukocyte-depletion [8–10]. These techniques could not decrease the volume of whole blood required for the measurements to less than 40 mL. Toyama *et al.* succeeded in decreasing the required volume to 20 mL, but they did not provide firm evidence on efficient removal of leukocytes from platelet suspension (see Supplementary Table S1) [11]. The aims of our study were: (1) to find a simple procedure for the separation of platelets from other blood cells, (2) to develop a method that requires only low volume of blood for mRNA quantification, and (3) to select and validate the most stably expressed mRNAs from a panel of seventeen RGs which can be used in RT-qPCR experiments for the normalization of transcript level in platelets of healthy individuals and patients with the history of myocardial infarction. The expression stability of the RGs was tested by four different approaches described in the literature [4,12–14] using RefFinder algorithm [15]. To ensure experimental transparency, accuracy, and repeatability, we followed the MIQE guidelines [16].

## 2. Results

### 2.1. Evaluation of RNA Integrity, Contamination and Inhibition

Amplification of each of the candidate genes was confirmed by the appearance of a single peak in the RT-qPCR melting curve analyses. Prior to carrying out RT-qPCR reactions, the integrity of all RNA samples was examined using real-time PCR to evaluate the expression of the *GAPDH* gene [5]. RNA integrity was in the acceptable range for all samples. When SPUD amplicons were added to each qPCR reaction in equal amount, the reactions demonstrated complete absence of qPCR inhibition. White blood cell contamination for all samples was ruled out by negative results (Cq > 40) of real-time PCR for granulocyte-specific mRNA (CD15) and lymphocyte-specific mRNA (HLA-DQβ).

### 2.2. Expression Level of Putative Reference Genes

*B2M*, *TBP*, *UBC*, *HMBS*, *PTMA*, *WIPI2*, *NCOA*, and *VAMP* were eliminated from further analyses during optimization. Exclusion criteria were unacceptable efficiency, low expression level, and the presence of non-specific products or primer dimer. Non-specific amplification and primer dimer can falsely increase transcript level, especially when intercalating dyes are used to assess real-time PCR.

**Figure 1:**
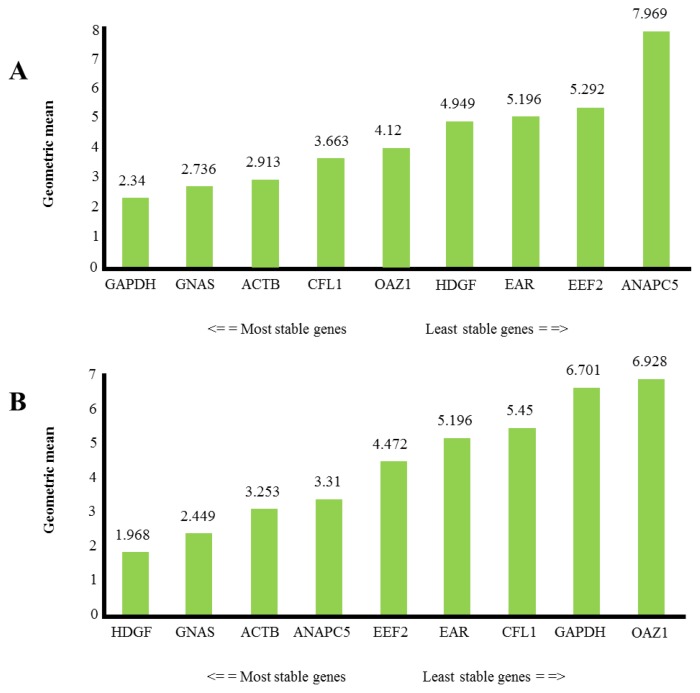
Comprehensive gene stability in healthy individuals (**A**), and in patients with the history of myocardial infarction (**B**).

Figure 1 shows the stability of reference genes for platelet transcript level study in healthy individuals and in patients with the history of myocardial infarction. Stability is expressed as the geometric means of results from four different types of calculation. Candidate reference genes showed Cq values between 19 and 34. The amplification efficiency of analyzed genes was as follows: *CFL1* (102%), *ACTB* (108%), *EEF2* (89%), *HDGF* (81.6%), *GAPDH* (117.3%), *ANAPC5* (81%), *GNAS* (83%), *EAR* (104%), *OAZ1* (96%) (Supplementary Table S2). According to Vandesompele *et al.*, minimum three internal control genes should be used for the correct normalization of RT-qPCR data. We found *GAPDH*, *GNAS*, and *ACTB* to be the best combination of reference genes for platelet transcript level studies in healthy individuals, while *HDGF*, *GNAS*, and *ACTB* are the best for studies in patients with the history of myocardial infarction.

**Figure 2:**
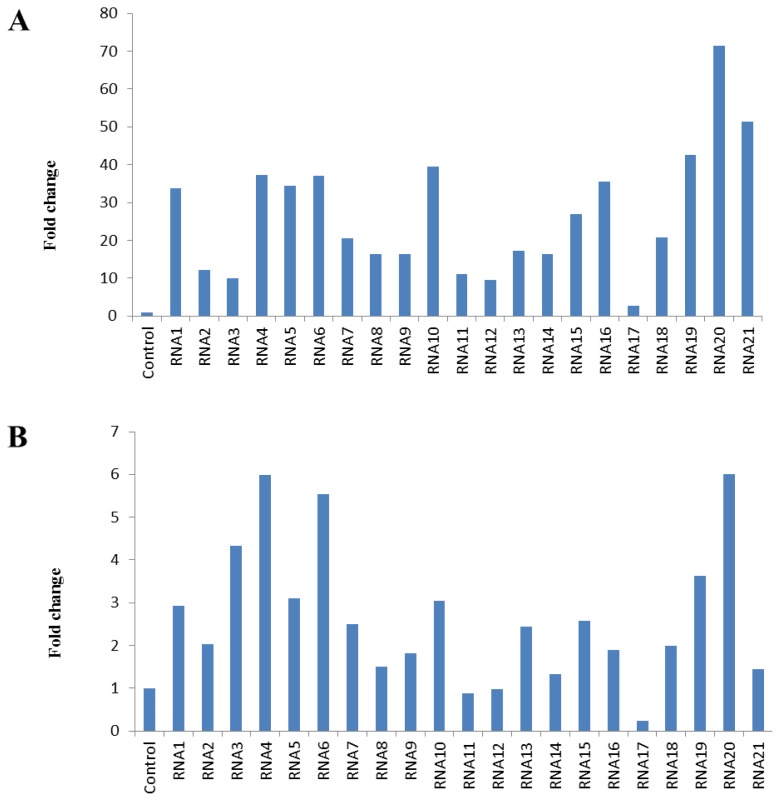
*Cyclo-oxygenase 1* (*COX-1*) transcript level in platelets of patients with the history of myocardial infarction. Transcript levels were normalized for *OAZ1* (**A**), or for *HDGF*, *GNAS*, and *ACTB* (**B**).

To demonstrate the usefulness of validated candidate reference genes in RT-qPCR, the expression level of *cyclo-oxygenase 1* (*COX-1*) gene transcript in platelets of patients with the history of myocardial infarction, relative to its expression in a control, was investigated, using the least stable gene (*OAZ1*) and the three most stable reference genes for normalization (Figure 2A,B). *COX-1* is constitutively expressed in many tissues and is responsible for physiological prostanoid production. The mean relative level of *COX-1* mRNA in platelets normalized for *OAZ1* was 10-fold higher than that normalized for *HDGF*, *GNAS*, and *ACTB*.

## 3. Discussion

A few methods have been published for the preparation of platelets free of contaminating leukocytes to study mRNA expression. These methods usually need high blood volume, and time-consuming preparation steps. In some cases, the absence of leukocytes from the final platelet suspension was not, or was not adequately verified. Our method of platelet isolation consists of three centrifugations (45 min) and needs only 12 mL of peripheral blood. Such platelet preparations were free of granulocytes and lymphocytes, as it was demonstrated by PCR-based methods.

The RT-qPCR is accepted as the method of choice for the accurate and sensitive quantification of transcript levels. The determination of reliable reference genes is an essential step for analyzing transcript level using RT-qPCR. Therefore, it is necessary to validate the expression stability of the control genes for specific organs, patient groups and/or experimental conditions prior to its use for normalization. It is a common practice to use traditional reference genes as internal controls without verifying their validity. Based on previous studies we investigated seventeen potentially reference genes and on the basis of optimizing studies, nine genes were selected for further studies. The stabilities of *EEF2*, *EAR*, *ACTB*, *GAPDH*, *ANAPC5*, *OAZ1*, *HDGF*, *GNAS*, and *CFL1* genes were determined by four different descriptive statistics.

Many analytical programs have been developed to correct sample-to-sample variation in studies for the identification of RGs and the relative quantification of transcript level in a study population. The use of multiple reference genes is the most accurate method. BestKeeper, NormFinder and geNorm programs are usually preferred, because they are supported by user-friendly software. The major weakness of geNorm and the approach developed by Silver *et al.* [13] are their sensitivity to co-regulation. Pairwise comparison apparently tends to select those genes with the highest degree of similarity in their expression profile. NormFinder approach does not account for systematic errors during sample preparation. The BestKeeper program considers all genes showing a variation in their amount of starting material by the factor two or more as unstable, which might limit its use [17].

It has been reported that three software programs (geNorm, NormFinder and BestKeeper) resulted in different ranking order in an attempt to select the most stable reference genes [17,18]; we also obtained different results using four different approaches (Supplementary Figures S1 and S2). It is a common practice to apply more than one statistical method for the selection of the most stable reference genes [18–20]. We employed the RefFinder program, which uses the mean of four statistical approaches and ranked the reference genes as a function of their means. Reference genes with the lower means were considered more stable than the others.

We acknowledge certain limitations of our study. Although a number of measures were introduced to keep platelet activation and RNA degradation at a minimum level, the maximum four-hour storage/transportation time might be a source of variability in certain conditions. When a study is carried out on samples of patients, it is difficult to minimalize the time between blood drawing and sample processing. In the case of different disease conditions the effect of storage/transportation on the integrity of platelet RNA should be evaluated. Another limitation of the study could be the age difference between the group of healthy individuals and patients with coronary artery disease. Although, in contrast to some other tissues, in whole blood no age dependence of gene expression was observed [21], age difference might introduce a certain level of uncertainty.

## 4. Experimental Section

### 4.1. Subjects

Twenty-one patients (age 51–77, 69% male) with the history of myocardial infarction were recruited for the study. The diagnosis of myocardial infarction was established at the time of its onset according to the criteria of the American College of Cardiology and the European Society of Cardiology. Patients were assessed for coronary sclerosis by coronary angiography. All patients demonstrated ≥50% stenosis at least in one major coronary artery or in one of their branches. Patients were on preventive low dose aspirin therapy. Platelets were also collected from seven apparently healthy individuals (age 26–54, 60% male) that did not take any medication.

### 4.2. Sample Collection and Preparation

Twelve mL blood samples were collected in tubes containing acid-citrate-dextrose (Becton Dickinson, Schwechat, Austria) The tubes were kept at ambient temperature and transported within four hours from the clinical ward to the analytical laboratory where it was immediately processed. On reaching the laboratory prostaglandin E1 (100 nmol/L) was added to each tube, and samples were centrifuged twice at 150*g*, for 15 min, at 37 °C. The rotor was allowed to decelerate without using the brake. Each time the upper 2/3 of the supernatant was used for the next step. After a third centrifugation (2500 rpm, 15 min, 37 °C) the supernatants were completely removed. It is advisable to invert the tubes on a sterile tissue for one minute and then wipe out their walls from supernatant’s remnants. Centrifugation is an inevitable step for platelet isolation, but it could activate platelets. Special care was taken to prevent platelet activation, which might have increased RNA degradation. Due to its low pH, anticoagulation by acid-citrate-dextrose, deceleration of centrifuges without brake, maintenance of temperature at 37 °C during platelet preparation, and the addition PGE1, an effective inhibitor of platelet activation all contributed to keeping platelet activation at a minimum level. It is to be noted that in preliminary experiments we did not observe any significant RNA degradation caused by up-to four hours storage/transportation and platelet preparation.

RNA was isolated from the platelet pellet by QIAamp RNA Blood Mini Kit (Qiagen, Hilden, Germany) according to the manufacturer’s instructions. As measured by NanoDrop 2000 spectrophotometer (Thermo Fisher Scientific, Waltham, MA, USA) the RNA concentrations were in the range of 2–4 ng/μL. However, as NanoDrop does not measure RNA concentration below 5 ng/μL precisely, we did not assign RNA concentration to the samples and in the case of all samples the same volume of RNA preparation was used for cDNA synthesis. The integrity of all RNA samples was examined by determining the *GAPDH* 3′:5′ signal ratio [5]. The signal ratio was around one (range: 0.96–1.02) demonstrating the high extent of integrity. SPUD assay was used to check for inhibition in each qPCR [22]. PCR analysis of each RNA sample was conducted to ensure the absence of RNA contamination from white blood cells [23]. All participants provided informed consent; the study was approved by the Ethical Committee of the Medical and Health Science Center, University of Debrecen.

### 4.3. Optimizing Primer Concentrations and PCR Efficiency

Sixteen non-ribosomal candidate reference genes were selected from lists of genes recommended as reference genes in two big studies [3,24]. In addition the Alu repeats were also evaluated as candidate reference gene [25]. Primers’ lengths were 19–25 nucleotides (TIB MOLBIOL, Berlin, Germany), with a theoretical *T*_m_ of 59–61 °C. The size of PCR amplicons ranged from 64–150 base pairs. Primers were designed to yield products spanning exon-exon boundaries to prevent possible amplification from contaminating genomic DNA. PCR products were subsequently resolved in 2% agarose gel to check for specific size of the amplicon. For gel electrophoresis, samples were resolved at 80 V in a 2% agarose gel with 0.5× Tris-borate/EDTA buffers and stained with ethidium bromide to visualize products. The individual efficiency for each primer pair were obtained by using standard curve [26]. As indicated by Pestana *et al.* and the manufacturer’s manual, the efficiency between 50% and 120% was accepted [27,28].

Our optimization goals were to identify the lowest primer concentration that still yields the lowest quantification cycle (Cq), results in maximum fluorescence and generates a single amplicon of correct size with predicted melting temperature. In these experiments, all combinations of six concentrations (100, 200, 300, 400, 600, 900 nM) of forward and reverse primers for seventeen genes were used to generate optimal amplification plots (Supplementary Table S2) [29]. These seventeen genes were: *GNAS* (guanine nucleotide-binding protein, alpha-stimulating), *ACTB* (actin, beta), *HDGF* (hepatoma-derived growth factor), *PTMA* (prothymosin, alpha), *B2M* (beta-2-microglobulin), *GAPDH* (glyceraldehyde-3-phosphate dehydrogenase), *HMBS* (hydroxymethyl-bilane synthase), *TBP* (TATA box binding protein), *UBC* (Ubiquitin C), *EAR* (expressed Alu repeats), *OAZ1* (ornithine decarboxylase antizyme 1), *WIPI2* (WD repeat domain, phosphoinositide interacting 2), *NCOA4* (nuclear receptor coactivator 4), *EEF2* (eukaryotic translation elongation factor 2), *VAMP* (vesicle-associated membrane protein), *ANAPC5* (anaphase promoting complex subunit 5), and *CFL1* (cofilin 1).

Primer and probe characteristics are shown in Supplementary Table S2. PCR efficiency (*E*), coefficients of determination (*R*^2^), and slope values were determined (Supplementary Table S2) using five serial 2-fold dilution points; Cq(s) were plotted *versus* the logarithm of dilution [26]. Minus RT controls, that were included for each run, were uniformly negative. PCR specificity for each gene was determined by dissociation curve analysis and gel electrophoresis. All primer sets produced a symmetrical amplicon peak in melting point analyses. In none of the samples was primer-dimer peak and no-template control (NTC) reaction (a negative control without cDNA template) was observed. The absence of primer-dimer was also verified by gel electrophoresis.

### 4.4. Reverse Transcription

RT was carried out under RNase-free conditions. Limited quantities of RNA dictated us to use fixed volume (5 μL) of input RNA for each cDNA synthesis [2]. RNA was reverse-transcribed to cDNA in 20 μL volume in the LightCycler 480 (Roche) by 1st Strand cDNA Synthesis Kit (Roche). For the subsequent RT reaction, 0.8 μg (0.04 *A*_260_ units) oligio-p [dT]_15_ primer, 1.6 μg (0.08 *A*_260_ units) random primer p[dN]_6_, 20 units AMV reverse transcriptase (Roche) and 50 units RNase inhibitor were added and incubated at 25 °C for 10 min and then at 42 °C for 60 min. We performed qPCR on RT triplicates instead of qPCR technical replicates [30].

### 4.5. qPCR

Real-time PCR using LightCycler 480 SYBR Green I Master (Roche) was performed in the LightCycler. PCR reaction consisted of 10 μL Master Mix (2× concentration), different concentration of primers as established by optimization (Supplementary Table S2), and 5 μL 10-fold diluted reverse transcribed total RNA (in a final volume of 20 μL). The following amplification program was used: heating for 10 min at 95 °C, 40 cycles of denaturation for 10 s at 95 °C, followed by 60 °C for 30 s (55 °C in case of GAPDH) and 72 °C for one second. Subsequently, a dissociation curve (melting curve) analysis was applied with one cycle at 95 °C for 15 s, 60 °C for 1 min and 0.5 °C ramp rate to 95 °C to confirm specific amplification. Cq-values were corrected for PCR efficiencies with the equation, Cq100% = CqLog*E*, (Cq100% = Cq at 100% efficiency). This way, the differences in efficiency between different reference genes were compensated.

### 4.6. Data Analysis

During the assessment of a set of RGs, the implemented evaluation method can be a source of bias related to the assumptions underlying each approach. In an effort to minimize bias, we tested the expression stability of nine selected internal control candidate genes by four different approaches found in the literature [4,12–14] by using RefFinder program [15].

ΔCq approach introduced by Silver *et al.* compares the relative expression of all pairwise combination of genes within each sample [13]. This comparison provides information on which pairs show least variability and hence which gene(s) has the most stable expression. The BestKeeper software calculates reference gene’s standard deviation (SD) based on raw Cq values regardless of sample’s efficiency [14]. NormFinder analysis enables estimation of the overall variation of the candidate normalization genes [12]. The combined measure of intra- and intergroup-variation is given as a stability value, which is an estimation of the variation in the expression of candidate RGs. The basic assumption is that a stable RG should have minimal variation across experimental groups and subgroups. Finally, geNorm is a Visual Basic Application for Microsoft Excel, which uses an algorithm to calculate *M*-value, a transcript level stability measure, defined as the mean pairwise variation for a given gene compared to the remaining tested genes. Stepwise exclusion of the reference gene with the least stable expression finally assigns the two most stable genes [4]. RefFinder is a web-based tool to select the most stably expressed mRNAs among a panel of RGs. It integrates geNorm, Normfinder, BestKeeper, and the comparative ΔCt method to rank the candidate RGs. It calculates the RG ranking based on each program, then evaluates final ranking by assigning a suitable weight to an individual gene and calculates the geometric mean of their weights.

## 5. Conclusions

According to our knowledge, this is the first study on the expression stability of candidate reference genes in platelets. This study identified groups of genes suitable for accurate normalization of RT-qPCR data in the sample of healthy individuals or patients with the history of myocardial infarction. Our results indicate that *GAPDH*, *GNAS*, and *ACTB* are the most stable genes expressed in platelets of healthy individuals and *HDGF*, *GNAS*, and *ACTB* were identified as the most stable reference genes expressed in platelets from patients with the history of myocardial infarction. The results on the expression of *COX-1* mRNA clearly demonstrated that the selection of inappropriate RG could lead to false assessment of the level of a specific transcript. The selection of appropriate RGs is essential for platelet transcript level studies.

## Supplementary Files

Supplementary File 1 Supplementary Information (PDF, 162 KB)
