# Supplementary material for: Validation of Reference Genes for the Determination of Platelet Transcript Level in Healthy Individuals and in Patients with the History of Myocardial Infarction
Source: Int J Mol Sci. 2013 Feb 6;14(2):3456–66. doi: 10.3390/ijms14023456 (PMC3588052; doi:10.3390/ijms14023456)
Supplement: Supplementary File 1 — Supplementary Information (PDF, 162 KB) [file ijms-14-03456-s001.pdf]

Supplementary Information

Table S1. Comparison of different platelet isolation methods.

Method	Blood volume	Important steps	Contamination check
Rox *et al*. [8]	40 mL	Filtration, exponential PCR amplification	Yes
Toyama *et al*. [11]	20 mL	Centrifugation	No
Amisten *et al*. [9]	100 mL	Filtration, antibody-mediated magnetic depletion	Yes
Amisten *et al*. [10]	<100 mL	Filtration, antibody-mediated magnetic depletion	Yes
Our method	12 mL	Centrifugation	Yes

Figure S1. Results of four different approaches used to validate reference genes for platelet transcript level determination in healthy individuals: Delta CT [13], Bestkeeper [14], Normfinder [12], and geNorm [4].

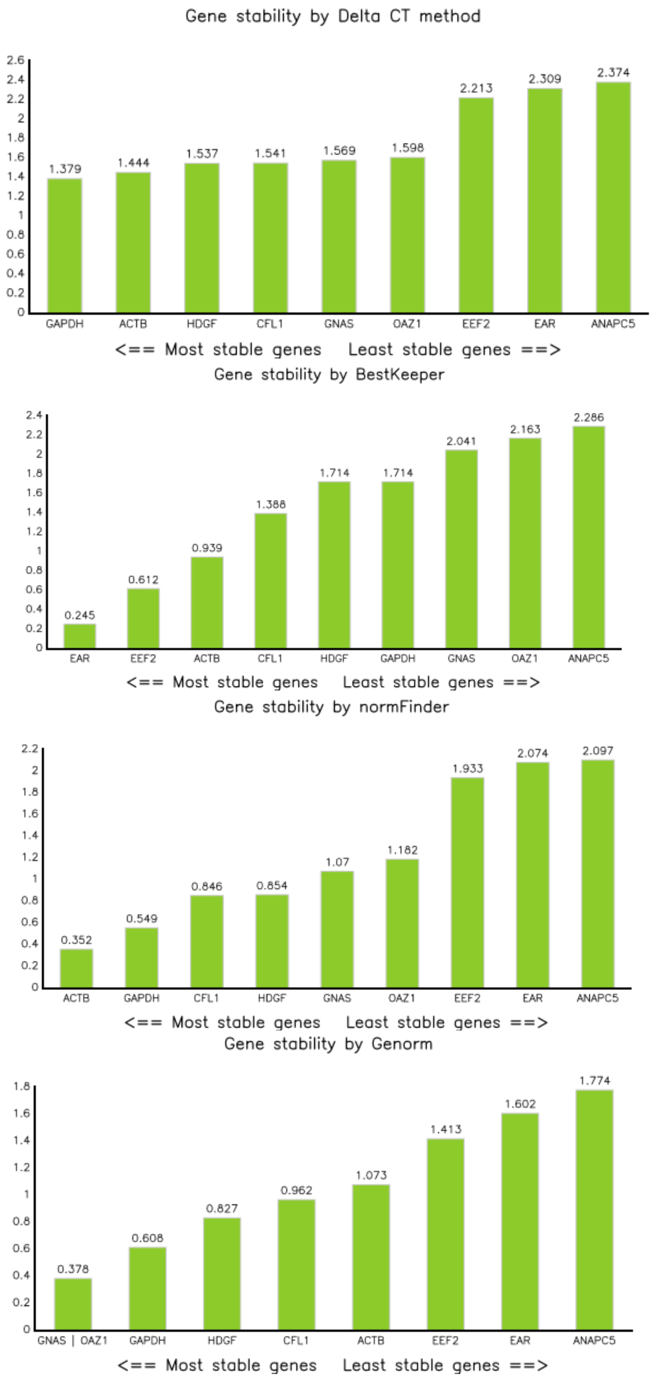

Table S2. Characteristics of qPCR primer pairs.

Gene symbol	Gene name	GenBank number	Primer sequence (5'–3')	Primer conc. (nM)	Amplicon size (bp)	qPCR efficiency (%)	R^2	Chromosome position	gDNA length (bp)
ACTB	Actin, beta	NM_001101	F: caaccgcgagaagatgac<br>R: gtccatcacgatgccagt	300	121	108	0.993	7p15–p12	551
ANAPC5	Anaphase promoting complex subunit 5	NM_016237	F: ttctgctggagcattctgtg<br>R: tctgttgaacaagggactgtattc	600	87	81	0.998	12q24.31	2270
B2M	Beta-2-microglobulin	NM_004048	F: tgccgtgtgaaccatgtga<br>R:ccaaatgcggcatcttcaa	900	98	96.6	0.999	15q21–q22.2	1975
CFL1	Homo sapiens cofilin 1	NM_005507	F: gtgccctctccttttcgttt<br>R: ttgaacaccttgatgacaccat	600<br>300	75	102	0.993	11q13	1929
EAR	Expressed Alu repeats		F: gaggctgaggcaggagaatcg<br>R: gtcgcccaggctggagtg	300	87	104	0.985		NA
EEF2	Eukaryotic translation elongation factor 2	NM_001961	F: ctggagatctgcctgaagga<br>R: gagacgaccgggtcagatt	600	74	89	0.984	19pter–q12	1230
GAPDH	Glyceraldehyde-3-phosphate dehydrogenase	NM_002046	F: tcaacgaccactttgtcaagc<br>R: ccaggggtcttactccttgg	200	109	117.3	0.991	12p13	255
GNAS	GNAS complex locus	NM_000516	F: aaggacaagcaggtctaccg<br>R: ggtgcttttaccagattctcca	300	72	83	0.997	20q13.3	3818
HDGF	Hepatoma-derived growth factor	NM_004494	F: ggagagcaggggacttgc<br>R: cctccttctcctctccttcag	600	78	81.6	0.998	1q21-q23	362
HMBS	Hydroxymethyl-bilane synthase	NM_000190	F: ccagctccctgcgaagag<br>R: ttccccgaatactcctgaactc	*	72	**	**	11q23.3	NA
NCOA4	Nuclear receptor coactivator 4	NM_005437	F: ctggtgagtcggtgacctg<br>R: tgccactctggtcttggaa	600<br>900	70	59.4	0.934	10q11.2	6704
OAZ1	Ornithine decarboxylase antizyme 1	NM_004152	F: ggatcctcaatagccactgc<br>R: tacagcagtggagggagacc	600	150	**	**	19p13.3	1780

Table S2. *Cont.*

Gene symbol	Gene name	GenBank number	Primer sequence (5'–3')	Primer conc. (nM)	Amplicon size (bp)	qPCR efficiency (%)	R^2	Chromosome position	gDNA length (bp)
OAZ1-r	Ornithine decarboxylase antizyme 1	NM_004152	F: caccatgccgctcctaag<br>R: gagggagaccctggaactct	900	67	96	0.993		1707
PTMA	Homo sapiens prothymosin, alpha	NM_002823	F:cctgctaacgggaatgctaa<br>R:cttcctcttcttcgtctacctca	*	73	**	**	2q35–q36	NA
TBP	TATA box binding protein	NM_003194	F:tgaatcttggttgtaaacttgacc<br>R: ctcatgattaccgcagcaaa	400	94	90	0.919	6q27	2379
UBC	Ubiquitin C	NM_021009	F: tcgcagccgggatttg<br>R:gcattgtcaagtgacgatcaca<br>Probe:FAM-tcgcagttcttgttgtg-BHQ1	900<br>200	64	141.8	0.811	12q24.3	876
UBC-r	Ubiquitin C	NM_021009	F: aggcaaagatccaagataagga<br>R: ggaccaagtgcagagtggac	900	132	176	0.820	12q24.3	162
VAMP	Vesicle-associated membrane protein	NM_003574	F: tgccagttatcacacgaagg<br>R: gaacagctttgctaggttcca	600<br>300	92	**	**	18p11.22	5428
WIPI-2	WD repeat domain, phosphoinositide interacting 2	NM_015610	F: tcatccccaacgagacttg<br>R: ggtgctcgctgtcctcat	300<br>600	106	**	**	7p22.1	1202

NA: not applicable; * PCR reaction could not be optimized; ** non-acceptable efficiency; Primer pairs for GNAS, ACTB, HDGF, PTMA, TBP, UBC-r, WIPI2, NCOA, EEF2, VAMP, ANAPC5, OAZ1-r, and CFL1 were designed using web-based Universal ProbeLibrary software [31]; Primers described in former publications were used for GAPDH [5], B2M (RTPrimerDB ID: 1234) [32], HMBS (Primer3 Plus) [33], UBC (RTPrimerDB ID: 7750), EAR [25], and OAZ1 [3]; In the cases of OAZ1 and UBC, due to the optimal PCR failure with the primers of the first design, second primer pairs were designed and used in the reactions.

Figure S2. Results of four different approaches used to validate reference genes for platelet transcript level determination in patients with the history of myocardial infarction: Delta CT [13], Bestkeeper [14], Normfinder [12], and geNorm [4].

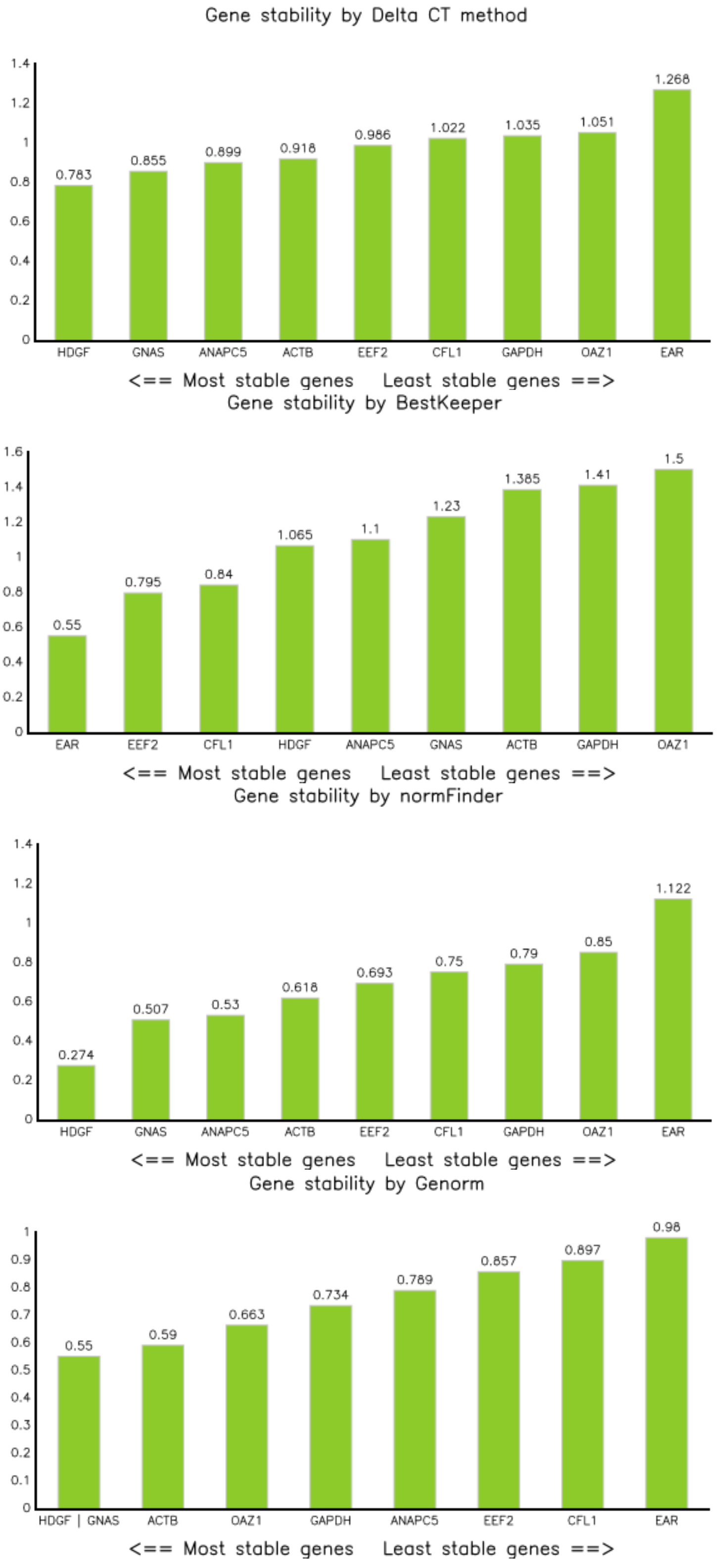

© 2013 by the authors; licensee MDPI, Basel, Switzerland. This article is an open access article distributed under the terms and conditions of the Creative Commons Attribution license (http://creativecommons.org/licenses/by/3.0/).
